# Patient distrust in pharmaceutical companies: an explanation for women under-representation in respiratory clinical trials?

**DOI:** 10.1186/s12910-020-00509-y

**Published:** 2020-08-13

**Authors:** Laurie Pahus, Carey Meredith Suehs, Laurence Halimi, Arnaud Bourdin, Pascal Chanez, Dany Jaffuel, Julie Marciano, Anne-Sophie Gamez, Isabelle Vachier, Nicolas Molinari

**Affiliations:** 1 Aix Marseille Univ, APHM, Hôpital NORD, CIC 9502, Clinique des bronches allergies et sommeil, Chemin des Bourrely, 13015 Marseille, France; 2 Aix Marseille Univ, CNRS, EFS, ADES, Marseille, France; 3 Aix Marseille Univ, INSERM U1263, INRA 1260 (C2VN), Marseille, France; 4 Department of Respiratory Diseases, Univ Montpellier, CHU Montpellier, Montpellier, France; 5 PhyMedExp, Univ Montpellier, CNRS, INSERM, CHU Montpellier, Montpellier, France; 6 Association pour l’Assistance et la Réhabilitation à Domicile (APARD), Montpellier, France; 7 Polyclinique Saint-Privat, Maladies Respiratoires et Troubles Respiratoires du Sommeil, Boujan sur Libron, France; 8 Clinique Générale de Marignane, Marignane, France; 9 IMAG, CNRS, Univ Montpellier, CHU Montpellier, Montpellier, France

**Keywords:** External validity, Clinical trials, Patients’ distrust, Recruitment bias, Under-representation of women

## Abstract

**Background:**

Patient skepticism concerning medical innovations can have major consequences for current public health and may threaten future progress, which greatly relies on clinical research.

The primary objective of this study is to determine the variables associated with patient acceptation or refusal to participate in clinical research. Specifically, we sought to evaluate if distrust in pharmaceutical companies and associated psychosocial factors could represent a recruitment bias in clinical trials and thus threaten the applicability of their results.

**Methods:**

This prospective, multicenter survey consisted in the administration of a self-questionnaire to patients during a pulmonology consultation. The 1025 questionnaires distributed collected demographics, socio-professional and basic health literacy characteristics. Patients were asked to rank their level of trust for pharmaceutical companies and indicate their willingness to participate in different categories of research (pre or post marketing, sponsored by an academic institution or pharmaceutical company).

Logistic regression was used to determine factors contributing to “trust” versus “distrust” group membership and willingness to participate in each category of research.

**Results:**

One thousand patients completed the survey, corresponding to a response rate of 97.5%. Data from 838 patients were analyzed in this study.

48.3% of respondents declared that they trusted pharmaceutical companies, while 35.5% declared distrust. Being female (*p* = 0.042), inactive in the employment market(*p* = 0.007), and not-knowing the name of one’s disease(*p* = 0.010) are factors related to declared distrust. Distrust-group membership is associated with unwillingness to participate in certain categories of trials such as pre-marketing and industry-sponsored trials.

**Conclusion:**

Distrust in pharmaceutical companies is associated with a specific patient profile and with refusal to participate in certain subcategories of trials. This potential recruitment bias may explain the under-representation of certain categories of patients such as women in pre-marketing drug trials.

## Background

Public distrust in healthcare systems and directed towards physicians, regulatory authorities and the pharmaceutical industry in general has increased over the past decades [1, 2]. A succession of health-related controversies and scandals related to safety issues involving pharmaceutical companies’ or physicians’ conflicts of interest has led to this erosion of trust [3]. The diffusion of selected information via mass media, which tends to sensationalize negative phenomena, greatly shapes the public’s perception of healthcare systems. The influence on medical decision-making among both physicians and patients is difficult to assess [4–7]. Pharmaceutical companies are suspected of putting profits above public interest, using marketing techniques to distort scientific evidence, and actively influencing both physicians and health policy makers [8–10].

This weak level of trust translates into skepticism about using pharmaceutical products, thus leading to new patient behaviors ranging from poor adherence [11, 12] to strong rejection of health policies, such as vaccine campaigns [13, 14]. Rejecting the implementation of medical and scientific findings not only has major consequences for current public health, but may also threaten future innovations and advances in medicine that principally rely on clinical research led by pharmaceutical companies, who have ever-growing needs for enrollment [15]. For clinicians, it can be fascinating to observe whether or not patients who have waited several weeks in order to attend a highly-specialized clinic for improving their condition are equally willing (or not) to participate in a clinical trial. In some countries, financial issues may affect this decision, but in the French health care system, all patients freely access all available drugs for a condition. One of the main motivations for attending a highly-specialized clinic in France is therefore the possibility of early access to new drugs or devices via participation in clinical research.

The recruitment and retention of patients in clinical trials is challenging, but necessary, because it is the cornerstone of medical evidence production. The additional challenge is to recruit a panel of patients as representative as possible of the future target population. The latter is meant to ensure the accuracy and generalizability of the efficacy and safety conclusions generated by clinical trials.

Nevertheless, the representativeness of study populations vis-à-vis real-life patients has been demonstrated as quite poor due to the narrow eligibility criteria required to enter a clinical trial [16]. Excessive eligibility criteria are a patient-extrinsic factor that can damage a trial’s external validity. In contrast, patient-determined factors may also apply, leading to the non-inclusion of seemingly otherwise eligible patients. Moreover, for geographical or investigator-determined cognitive bias some patients are never approached by centers conducting clinical trials which may weaken even more the external validity of trials [17].

In this study, we hypothesized that distrust in pharmaceutical companies is correlated with a specific psychosocial profile among patients.

We sought to evaluate patient willingness to participate in different categories of clinical trials and associated predictive factors in order to determine whether or not global distrust in the healthcare system could represent a recruitment bias in clinical trials.

## Methods

### Study setting and population

This prospectively designed study was approved by an independent ethics committee (Le Comité de Protection des Personnes Sud-Méditerranée I; reference number: 14–72). As per French legislation and ethics committee approval, no written inform consent was required. Patients’ consent was implied upon the completion of the questionnaire. On 27 march 2017, study has been retrospectively registered on open science framework and www.clinicaltrials.gov with reference number NCT03098303.

Anonymous self-questionnaires were filled out before consultation by consecutive patients consulting for respiratory diseases (excluding oncology and tobaccology) in the respiratory disease departments of 4 respiratory centers in southern France between December 2014 and December 2016.

The participating establishments included two public (teaching/research) hospitals (The University Hospitals of Marseille and Montpellier) and two private centers (The Polyclinique Saint-Privat, Boujan-sur-Libron, and the Clinique de Marignane, Marignane).

### The questionnaire

The PROTOACCEPT questionnaire included fields corresponding to age, sex, employment, education level (university schooling or not), whether or not the participant knew the name of his/her disease (yes/no), whether or not he/she had already participated in a research protocol (yes/no/I don’t know) and whether or not this was his/her first consultation in the department (yes/no). The general diagnosis (interstitial lung disease, chronic airway disease, sleep disorders, other), indicated by the investigator, was also recorded for each patient, as well as the public or private nature of the including center.

Patients were also asked to rank their distrust for pharmaceutical company medical research studies according to a 5-point likert scale (“I would refuse participation in a medical research study because I distrust pharmaceutical companies”: Strongly disagree – Disagree – Neither agree nor disagree – Agree – Strongly agree). They were further asked whether or not they would agree to test a new drug before (yes/no) or after (yes/no) commercialization, and whether or not they would agree to participate in research at different kinds of institutions (pharmaceutical company and three types of public facilities: university hospital, general hospital, or a public research institute)(yes/no for each option).

This questionnaire was built by a multidisciplinary team (private and public physicians, psychologist, nurses, and a pharmacist) and validated by a psychologist in a 3 steps process. The first step included a qualitative pre-survey with thirty patients and consisting in open questions designed to capture events to report in the questionnaire. In the second step, a first version of the questionnaire was administered face to face by the psychologist to ten patients. Ambiguous words were removed and medical vocabulary simplified. The final version was tested on five patients in typical survey settings, in the presence of the psychologist but without her intervention.

### Group classification

Patients who indicated that they agreed or strongly agreed with the statement “I would refuse participation in a medical research study because I distrust pharmaceutical companies” defined the “distrust” group. Patients who checked one of the other three categories (neutral or disagreement) defined the “trust” group. Patients who did not answer the question were not classified.

### Sample size and missing data

The sample size was arbitrarily set at 1000 answered questionnaires. Variations in count data are due to missing data, which was consistently under 20% for all questionnaire fields and therefore not otherwise addressed.

### Statistics

All statistical analyses were carried out using the R (version 3.3.0) programming environment [18]. Results are presented as numbers and percentages for qualitative variables. After evaluating the distribution of the age variable via a Shapiro-Wilks test, the latter was presented via medians with interquartile ranges. Group comparisons were performed using unadjusted χ^2^ tests for qualitative variables and Mann-Whitney tests for age.

Multiple (logistic) regressions were performed with covariates (variables were selected if their associated *P* value was less than 0.15 in univariate analyses and a backward procedure was used to select the final model) and then presented as adjusted Odds Ratios with 95% confidence intervals. The first model performed used age, gender, education, employment, doctor-reported diagnosis group, patient experience (first consult in respiratory disease ward, previous participation in research), the public/private nature of the including center and whether or not the patient knew the name of his/her pathology to predict patient membership in “trust” versus “distrust” groups. To additionally explain patient willingness to participate in new-drug studies or research associated with pharmaceutical companies or with public institutions, further models used the same predictive variables as for the first model, plus distrust-group-membership as an additional explanatory variable.

## Results

### Trust and distrust group descriptions

One thousand questionnaires were answered (the response rate to this questionnaire was 97.5%.), 355 (35·5%) patients were in the “distrust” group, 483 (48·3%) in the “trust” group, and 162 (16·2%) did not answer the question (unclassified). Thus, our analysis was performed on 838 patients characterized by their membership in the “trust” or “distrust” groups. As compared with the “trust” group, individuals in the “distrust” group were slightly but significantly older and significantly more often professionally inactive (Table 1). The two groups were statistically similar in terms of patient experience, be it as concerns previous consults in the respiratory disease ward, whether or not the latter was public or private, or as concerns previous participation in clinical research. Despite similar rates of university-level education, the distrust group had significantly fewer patients who knew the name of their pathology, and fewer patients with a doctor-reported diagnosis relating to a sleep disorder (Table 1).

**Table 1:** Population characteristics for the “Trust” and “Distrust” groups. *P* values correspond to between-group comparisons (either a Mann-Whitney test for Age, or χ^2^ tests for categorical variables)

	Distrust *N* = 355	Trust *N* = 483	*p*-value
Age, median [IQR]	61 [49–68]	57 [44–67]	< 0.001
Sex (Male, %)	161/354 (45)	251/483 (52)	0.064
University Yes	133/342 (39)	185/454 (41)	0.596
Employment:			
Active, not working	16/352 (5)	33/480 (7)	0.033
Active, working	130/352 (37)	208/480 (43)	
Inactive	206/352 (58)	239/480 (50)	
Diagnosis:			
Chronic Airway Disease	233/346 (67)	339/472 (72)	0.031
Interstitial lung disease	45/346 (13)	47/472 (10)	
Sleep disorders	33/346 (10)	59/472 (12)	
Other	35/346 (10)	27/472 (6)	
Has already participated in a clinical trial:			
Yes	57/348 (16)	83/476 (17)	0.402
No	258/348 (74)	360/476 (76)	
Unknown	33/348 (10)	33/476 (7)	
First consultation for respiratory disease	63/351 (18)	86/477 (18)	0.976
Inclusion center, public	257/355 (77)	362/483 (75)	0.406
The patient knows the name of his/her disease	259/348 (74)	388/477 (81)	0.017

### Factors associated with distrust group membership

In terms of selecting potential factors for predicting “distrust”, the following variables provided significant (or near-significant) results at the univariate level: age, gender, inactive employment status, doctor-reported sleep disorder, and whether or not the patient knew his/her pathology. Of the latter, only three variables remained significant at the multivariate level: in summary, being female, inactive in the employment market, and not knowing the name of one’s pathology are factors related to the patient’s declared distrust in pharmaceutical companies (adjusted odds ratios (aOR) of 1·33 [1·09–1·79; *p* = 0·042], 1·47 [1·11–1·94; p = 0·007] and 1·56 [1·11–2·17; p = 0·010], respectively) (Table 2).

**Table 2:** Multiple (logistic) regression results: crude (cOR) and adjusted (aOR) odds ratios with 95% confidence intervals [95%CI] and associated significance estimates (*p*-values) for variables used to predict distrust-group membership

	cOR [95%CI]	*p*-value	aOR [95%CI]	*p*-value
Age	1.01 [1.00–1.02]	0.040		
Gender = male	0.77 [0.59–1.02]	0.064	0.75 [0.56–0.92]	0.042
University = yes	0.92 [0.69–1.23]	0.596		
Employment = inactive	1.42 [1.08–1.88]	0.013	1.47 [1.11–1.94]	0.007
Diagnosis				
Chronic airway disease	Reference			
Interstitial lung disease	1.39 [0.90–2.17]	0.141		
Sleep disorders	0.81 [0.52–1.27]	0.019		
Other	1.89 [1.21–2.93]	0.377		
The patient knows the name of his/her disease	0.67 [0.48–0.93]	0.017	0.64 [0.46–0.90]	0.010
Has already participated in a clinical trial	1.08 [0.79–1.49]	0.625		
First consultation for respiratory disease	0.99 [0.69–1.42]	0.976		
Inclusion center = public	0.88 [0.64–1.20]	0.406		

### Patient willingness to participate in different clinical trial subcategories

93% (778/838) of surveyed patients declared their willingness to participate in at least one subcategory of trial. 76% (638/838) indicated that they were willing to test a drug (75% (630/838) if the drug was already on the market and 29% (246/838) prior to market authorization). A large majority (86%, 723/838) of patients were willing to participate in a study sponsored by a public institution, whereas only 11% (91/838) of patients indicated that they were willing to participate in a pharmaceutical-led study.

### Factors associated with patient willingness to test drugs

When using multivariate logistic regression models for predicting responses to whether or not patients agreed to test a drug either before or after commercialization, before only and after only, female gender appears to be associated with refusal (adjusted odds ratios (aOR) of 0.67 [0.49–0.93; *p* = 0.017], 0.64 [0.47–0.88; *p* = 0.006], and 0.66 [0.50–0.88; *p* = 0.004], respectively). Distrust group membership, however, was negatively associated with willingness to test drugs before commercialization (aOR: 0.45 [0.32–0.63; *p* <  0.001]), and had a similar tendency for the “before or after” model (aOR: 0.73 [0.53–1.01; *p* = 0.060]), but showed no significant relationship with willingness to test drugs after commercialization. Finally, ignoring the name of his/her disease is associated with unwillingness to test a drug before commercialization (aOR: 0.58 [0.39–0.88; *p* = 0.010]), but is otherwise not significant in the other drug-willingness models (Table 3).

**Table 3:** Factors associated with refusal to participate in clinical trial subcategories (adjusted (aOR) odds ratios with 95% confidence intervals [95%CI] and associated significance estimates (*p*-values) for variables used to predict refusal)

Trial Subcategory	Willingness to participate rate	Factors associated with refusal to participate	aOR [95%CI]	*p*-value
Drug Pre-marketing	246/838 (29%)	- Gender (female)	0.64 [0.47–0.88]	0.006
		- Poor knowledge about own disease	0.58 [0.39–0.88]	0.010
		- Distrust-group membership	0.45 [0.32–0.63]	< 0.001
Drug Post-marketing	630/838 (75%)	- Gender (female)	0.66 [0.50–0.88]	0.004
Drug (pre or post-marketing)	638/838 (76%)	- Gender (female)	0.67 [0.49–0.93]	0.017
Public sponsor	723/838 (86%)	- Inactive employment status	0.56 [0.40–0.78]	< 0.001
Pharmaceutical sponsor	91/838 (11%)	- Age	0.96 [0.95–0.98]	< 0.001
		- Distrust-group membership	0.25 [0.14–0.44]	< 0.001

### Factors associated with patient willingness to participate in research with pharmaceutical companies or public institutions

As concerns patient willingness to participate in research with a pharmaceutical company or at least one of three types of public institution, distrust-group membership and age were both negatively associated with pharmaceutical company research (aOR: 0.25 [0.14–0.44; *p* <  0.001] and 0.96 [0.95–0.98; *p* < 0.001], respectively). An inactive employment status was the only variable that significantly negatively contributed to willingness to participate in research organized by a public institution (aOR: 0.56 [0.40–0.78; *P* < 0.001]) (Table 3).

## Discussion

Our study aimed at evaluating variables associated with patient willingness to participate in different categories of clinical trials and at identifying a potential recruitment bias in clinical trials related to patient distrust in the pharmaceutical industry and healthcare systems.

For this purpose, we conducted a two-part analysis in respiratory disease patients surveyed about their opinions concerning clinical research and potential willingness to participate. The majority of patients surveyed suffered from chronic airway diseases (asthma, chronic obstructive pulmonary disease (COPD), i.e. the most prevalent chronic respiratory diseases in France [19].

First, we compared patient characteristics on the basis of their level of trust in the pharmaceutical industry. In a second step, we assessed willingness-to-participate in clinical trials for our whole population to identify factors associated with acceptance or refusal. Several studies have previously evaluated such rates and highlighted that altruism, hope for personal benefit, contribution to advances in science as well as financial benefit are the main reasons for agreeing to participate, whereas fear of adverse events, impossibility to cope with the logistic constraints accompanying participation, poor knowledge about or negative perception of clinical trials and distrust in pharmaceutical industry are potential barriers [20–23].

Thus, distrust in pharmaceutical companies was suspected to decrease patient willingness to participate in clinical trials but, to our knowledge, this has never been confirmed by juxtaposing patient-reported willingness-to-participate and their level of trust in pharmaceutical companies.

We found that 35.5% of respiratory disease patients declared they did not trust pharmaceutical companies. One major finding is that the profile of the latter, “distrusting” patients significantly differs from trusting patients: distrust is associated with female gender, inactive professional status and lack of knowledge on one’s own disease.

In the second step of our analysis, we calculated rates of willingness to participate in clinical trials. Such rates reported in medical literature are extremely heterogeneous [20–25]. This variation seems to depend on certain study characteristics: for example, willingness increases with disease severity [24]. Clinical research is a wide and heterogeneous area with multiple methodologies, interventions and objectives. Referring to “clinical trials” as a single concept is simplistic. Similarly, considering willingness-to-participate in a specific subcategory of clinical trials in order to assess global perceptions of clinical research may be misleading.

Because we distinguished different categories of clinical trials in our survey, we were able to identify trial-category-related barriers and associated differences in patient profiles. Thus, the overall rate of willingness-to-participate in at least one category of trial was very high (93%) among the 838 surveyed patients we analyzed. Globally, patients are not reluctant to join clinical research but, as expected, we found rates that vary significantly depending on the type of trial (intervention tested, market status of the drug and type of trial sponsor) ranging from 11 to 86%. Refusal of drug trials was associated with sex, women being more reluctant to join drug trials than men. Among drug trials, an important difference exists in patient willingness-to-participate between pre-marketing and post-marketing studies. Only 29% of patients we surveyed would have accepted enrolment in a pre-marketing drug trial, while 75% would have participated in a post-marketing trial. We assume this highlights patient trust in the regulatory health authorities responsible for marketing approval. Poor knowledge about one’s own disease and distrust in the pharmaceutical industry were associated with the refusal of pre-marketing drug trials, but played no role in refusing post-marketing trials.

When comparing willingness-to-participate among different types of clinical trial sponsors, we found consistent results with only 11% of patients considering participation in industry-sponsored trials, while 86% would have agreed if the sponsor were academic. Again, distrust in pharmaceutical companies plays a role in this difference. This is a major finding which, to our knowledge, has never been identified previously. It may also present a lever for external validity improvement in industry-sponsored trials. Thus, targeted educational programs for improving knowledge on both diseases and clinical trials, as well as unbiased media communications about the benefits and risks of collaboration with pharmaceutical companies, could restore patient trust in pharmaceutical companies and increase willingness-to-participate in clinical trials. A joint effort for education and communication involving mass media, the pharmaceutical company, regulatory authorities and physicians is crucial for both current and future public health.

Additionally, post-marketing academic trials should be encouraged as well as pooled analyses of industrial and academic results.

The main limitation of the present survey is that it was conducted on a specific patient population, i.e. chronic respiratory disease patients. The results should not be extrapolated beyond chronic diseases which are not life-threatening in the short term. The second specificity is that the patients surveyed, living in France, have access to a robust health system which covers the costs of medical care and treatment. In some countries, participation in a clinical trial may be the only way to access care and treatment. Our study does not analyze if the barriers to participation that we have identified are likely to influence decision-making in this context.

One other limitation of our survey was that we assessed the hypothetical intention to participate, which has been shown to be higher than real participation rates [25]. Some patients who declare they would accept participation may refuse once the offer to participate becomes real. We hypothesize that this situation may occur in any subgroup of patient and thus assume that the ranking of rates among trial subcategories, as well as refusal risk factors, are accurate.

The questionnaire was developed specifically in French for this study and has not been previously published in any peer-reviewed journal. A bilingual version, provided without cultural validation of the translation, is available as additional file.

In short, our results suggest that patient distrust in the pharmaceutical industry could help explain recruitment bias in industry─sponsored clinical trials conducted in similar settings. Attention should be paid to this phenomenon because the majority of therapeutic innovations are provided by pharmaceutical companies able to invest significant resources in research and development [15]. Moreover, we found that patient distrust is associated with a distinct patient profile. Thus, we have no guarantee of the applicability of trial results for these patients. The under-representation of women, minorities and patients with poor health literacy in industry-sponsored clinical trials has recently gained awareness [26–29] and may threaten the generalizability of results [28]. However, there is still very little available data focusing on women enrolment by country or disease. In the French asthma population for example, percentages of women enrolled are higher in women in France in a national academic interventional cohort than in a national industry led early access program to mepolizumab (64.5 and 45%, respectively) [30, 31]. Some data suggest that this under-representation of women exists worldwide even in countries where patients might be willing to participate in trials only to have their treatment free of charge [32, 33].

In this survey, we were not authorized to collect ethnicity, so we cannot conclude on this specific characteristic. However, we found that women and patients with poor knowledge concerning their disease were more likely to distrust pharmaceutical companies and are less willing to participate in industry-sponsored trials. This could at least partly explain underrepresentation of these sub-populations in industry-sponsored clinical trials and could be addressed by implementing educational strategies [34–36] as mentioned previously.

The publishing process of the present manuscript takes place during the COVID-19 pandemic period. It is difficult to anticipate how the current situation will shape and modify patients’ perception of pharmaceutical companies. Trusting opinions associated with the hope that pharmaceutical companies will find a treatment and distrusting opinions that accuse such companies of taking advantage of the situation to make money coexist. This should be the endpoint of a specific future study.

## Conclusions

Distrust in pharmaceutical companies is associated with a specific patient profile and with refusal to participate in pre-marketing industry-sponsored drug trials. Our results suggest that patient distrust in the pharmaceutical industry could help to explain recruitment bias in industry─sponsored clinical trials. Further studies are needed to extrapolate results to different healthcare systems and beyond chronic diseases.

A potential means for improving the external validity of industry-sponsored clinical trials is to implement educational strategies to increase unbiased knowledge of these trials. There are many ways to achieve this call to action. Any initiative is beneficial and all communication media, small and large scale, are complementary. A joint effort dedicated to fighting against fake news and biased media supported by the payers and policy makers appears mandatory and eventually protective.

## Supplementary information

**Additional file 1.** Questionnaire PROTOACCEPT - French/English bilingual version.

## Data Availability

The datasets used and/or analyzed during the current study are available from the corresponding author on reasonable request after approval from all the authors.
