# Supplementary material for: Patient distrust in pharmaceutical companies: an explanation for women under-representation in respiratory clinical trials?
Source: BMC Med Ethics. 2020 Aug 13;21:72. doi: 10.1186/s12910-020-00509-y (PMC7424561; doi:10.1186/s12910-020-00509-y)
Supplement: Supplementary file 1 — Additional file 1. Questionnaire PROTOACCEPT - French/English bilingual version. [file 12910_2020_509_MOESM1_ESM.zip › PROTOACCEPT1_bilingual_questionnaireR5.docx]

Madame, Monsieur,

Madame, Sir,

Nous aimerions que vous preniez quelques minutes pour remplir ce questionnaire portant sur la recherche médicale. Ce questionnaire comporte 4 pages, **il est anonyme** et il n’y a pas de bonnes ou mauvaises réponses.

We would like you to take a few minutes to complete this medical research questionnaire. This questionnaire has 4 pages, **it is anonymous** and there are no right or wrong answers.

| Age: …………….Ans  Age:……… ………….Years | Sexe: ⭘ Masculin ⭘ Féminin  Sex: Male Female |
| --- | --- |

Nombre de personnes vivant dans le logement : ………………….

Number of people living in your home/residence:

Votre niveau d’études atteint (cochez la case)

The highest level of education you reached (check one box)

| ⭘ Certificat d’études primaires  Primary school  ⭘ Collège, CAP, BEP  Middle school / Juniour High School  ⭘ Seconde, première ou terminale  High school | ⭘ Etude supérieures  University or equivalent  ⭘ Autre ……………………………………………..  Other |
| --- | --- |

Votre situation par rapport à l’emploi (cochez la ou les cases)

Your employment situation (check one or more boxes)

| 🞏 Indépendant(e) ou à votre compte  Independent or self-employed  🞏 Titulaire de la fonction publique  Civil servant  🞏 Emploi stable (CDI)  Stable employment (Permanent contract)  🞏 Emploi précaire (CDD, intérim, stage)  Precarious/short-term employment (fixed-term contract, interim, internship)  🞏 Demandeur d'emploi non indemnisé(e)  Non-compensated job seeker  🞏 Demandeur d'emploi indemnisé(e)  Job seeker receiving benefits | 🞏 Au foyer  Home-maker  🞏 Retraité(e)  Retired  🞏 Allocataire du RSA  Active Solidarity Income recipient  🞏 Allocation adulte handicapé  Disabled adult allowance  🞏 Allocation travailleur handicapé  Disabled worker allowance  🞏 Etudiant(e)  Student  🞏 Autre………………………………….  Other |
| --- | --- |

1. Connaissez-vous le nom de la maladie actuelle pour laquelle vous venez consulter ?

Do you know the name of the illness for which you are consulting today?

⭘ Oui ⭘ Non

Yes No

Si oui, quel est son nom ? ……………………………………..…………………………………….

If yes, what is its name?

Tournez la page SVP

Please turn the page

2. Est-ce votre première consultation pour votre maladie respiratoire ?

Is this your first consultation for your respiratory illness?

⭘ Oui ⭘ Non

Yes No

3. Avez-vous déjà participé à un protocole de recherche médicale ?

Have you ever participated in a medical research protocol?

⭘ Oui ⭘ Non

Yes No

4. Si un médecin vous proposait de participer à un protocole de recherche médicale, quelle serait votre première réaction ?

If a doctor suggested that you participate in a medical research protocol, what would be your first reaction?

⭘ Oui ⭘ Non ⭘ Je souhaiterai y réfléchir

Yes No I would like to think about it

5. Dans le tableau ci-dessous :

In the table below:

A - Veuillez d'abord tout lire, puis choisir et cocher 3 propositions qui vous semblent les plus caractéristiques de "La Recherche Médicale". Ne cochez pas plus de 3 cases.

Please read everything first, then choose and tick 3 proposals that seem to you the most characteristic of "Medical Research". Do not tick more than 3 boxes.

B - Puis, dans les propositions restantes, veuillez choisir et cocher 3 propositions qui vous semblent les moins caractéristiques de "La Recherche Médicale". Ne cochez pas plus de 3 cases.

Then, in the remaining proposals, please choose and tick 3 proposals which seem to you the least characteristic of "Medical Research". Do not tick more than 3 boxes.

(Merci de bien vouloir respecter la totalité de la consigne)

(Please respect all of the instructions)

| **La Recherche Médicale, c'est surtout…**  Medical Research is above all… | Les plus caractéristiques  Most characteristic | Les moins caractéristiques  Least characteristic |
| --- | --- | --- |
| … une possibilité pour les malades d'avoir les traitements les plus récents  … a possibility for patients to have the most recent treatments | 🞏 | 🞏 |
| … une affaire d'argent  … a matter of money | 🞏 | 🞏 |
| … une avancée de la science  … a way to make progress in science | 🞏 | 🞏 |
| … une utilisation des malades et de leurs maladies  … a way to use the sick and their illnesses | 🞏 | 🞏 |
| … un investissement économique pour un pays  … an economic investment for a country | 🞏 | 🞏 |
| … une prise de risque pour les malades et leur santé  … a risk to patients and their health | 🞏 | 🞏 |
| … une meilleure prise en charge médicale pour les malades  …better medical care for the sick | 🞏 | 🞏 |
| … un enjeu pour les laboratoires pharmaceutiques  … an issue at stake for pharmaceutical companies | 🞏 | 🞏 |
| … un espoir de guérison pour les malades  … hope of healing for the sick | 🞏 | 🞏 |
|  | Mettre 3 croix obligatoirement  You must tick three | Mettre 3 croix obligatoirement  You must tick three |

Tournez la page SVP

Please turn the page

6. Voilà une liste des raisons qui pourraient contribuer à votre acceptation à participer à une recherche médicale. Veuillez indiquer dans quelle mesure vous êtes d'accord ou pas d'accord sur toutes ces raisons en cochant la case appropriée.

Here is a list of reasons that may explain why you would accept to participate in medical research. Please indicate to what extent you

agree or disagree with all of these reasons by checking the appropriate box.

| J'accepterais parce que….  I would accept because…. | Pas du tout d'accord  Strongly disagree | Pas d'accord  Disagree | Ni d'accord ni pas d'accord  Neither agree nor disagree | D'accord  Agree | Tout à fait d'accord  Strongly agree |
| --- | --- | --- | --- | --- | --- |
| … c'est utile pour ma santé actuelle  … it’s useful for my current health | ⭘ | ⭘ | ⭘ | ⭘ | ⭘ |
| … j'ai envie de faire avancer la science  … I want to advance science | ⭘ | ⭘ | ⭘ | ⭘ | ⭘ |
| … ma famille m'incite à le faire  … my family encourages me to do it | ⭘ | ⭘ | ⭘ | ⭘ | ⭘ |
| … je vais voir mon médecin plus souvent  … I will see my doctor more often | ⭘ | ⭘ | ⭘ | ⭘ | ⭘ |
| … c'est indemnisé  … it’s compensated | ⭘ | ⭘ | ⭘ | ⭘ | ⭘ |
| … je vais me sentir mieux pris(e) en charge  … I will feel better taken care of | ⭘ | ⭘ | ⭘ | ⭘ | ⭘ |
| … j'ai envie de guérir  … I want to heal | ⭘ | ⭘ | ⭘ | ⭘ | ⭘ |
| … j'ai du temps  … I've got time | ⭘ | ⭘ | ⭘ | ⭘ | ⭘ |
| … c'est difficile de dire non à un médecin  … it's hard to say no to a doctor | ⭘ | ⭘ | ⭘ | ⭘ | ⭘ |
| … j'ai confiance dans mon médecin  … I trust my doctor | ⭘ | ⭘ | ⭘ | ⭘ | ⭘ |
| … c'est utile pour ma santé future  … it’s useful for my future health | ⭘ | ⭘ | ⭘ | ⭘ | ⭘ |
| … c'est utile pour ceux qui ont la même maladie que moi  … it’s useful for those who have the same illness as me | ⭘ | ⭘ | ⭘ | ⭘ | ⭘ |
| … quelqu'un de proche m'en a parlé positivement  ... someone close to me said positive things about it | ⭘ | ⭘ | ⭘ | ⭘ | ⭘ |

Tournez la page SVP

Please turn the page

7. Voilà une liste des raisons qui pourraient contribuer à votre refus de participer à une recherche médicale. Veuillez indiquer dans quelle mesure vous êtes d'accord ou pas d'accord sur toutes ces raisons en cochant la case appropriée.

Here is a list of the reasons that may explain why you would refuse to participate in medical research. Please indicate to what extent you

agree or disagree on all of these reasons by checking the appropriate box.

| Je refuserais parce que…  I would refuse because…. | Pas du tout d'accord  Strongly disagree | Pas d'accord  Disagree | Ni d'accord ni pas d'accord  Neither agree nor disagree | D'accord  Agree | Tout à fait d'accord  Strongly agree |
| --- | --- | --- | --- | --- | --- |
| … c'est inutile pour ma santé actuelle  … It's useless for my current health | ⭘ | ⭘ | ⭘ | ⭘ | ⭘ |
| … je n'aime pas aller à l'hôpital  … I don't like going to the hospital | ⭘ | ⭘ | ⭘ | ⭘ | ⭘ |
| … je n'aime pas aller chez le médecin  … I don't like going to the doctor | ⭘ | ⭘ | ⭘ | ⭘ | ⭘ |
| … je n'ai pas confiance  … I’m wary of the situation | ⭘ | ⭘ | ⭘ | ⭘ | ⭘ |
| … je me méfie des laboratoires pharmaceutiques  … I don’t trust pharmaceutical companies | ⭘ | ⭘ | ⭘ | ⭘ | ⭘ |
| … je n'aime pas les examens médicaux  … I don't like medical exams | ⭘ | ⭘ | ⭘ | ⭘ | ⭘ |
| … je n'ai pas trop le temps  … I don't have enough time | ⭘ | ⭘ | ⭘ | ⭘ | ⭘ |
| ...quelqu'un de proche m'en a parlé négativement  ... someone close to me said negative things about it | ⭘ | ⭘ | ⭘ | ⭘ | ⭘ |
| … j'habite trop loin  … I live too far away | ⭘ | ⭘ | ⭘ | ⭘ | ⭘ |
| … c'est difficile de venir  … It's difficult to come in | ⭘ | ⭘ | ⭘ | ⭘ | ⭘ |
| … je ne veux pas être un cobaye  … I don't want to be a guinea pig | ⭘ | ⭘ | ⭘ | ⭘ | ⭘ |
| … ça coûte cher de venir souvent  … It is expensive to come in often | ⭘ | ⭘ | ⭘ | ⭘ | ⭘ |
| … je risque d'avoir un placebo  … I risk getting a placebo | ⭘ | ⭘ | ⭘ | ⭘ | ⭘ |

Tournez la page SVP

Please turn the page

8. Quel temps pourriez-vous donner à une recherche médicale sur une période d’une année ? (Une seule réponse possible)

How much time could you give to medical research over a period of one year? (Only one answer possible)

| ⭘ 1 fois tous les 15 jours  Once every 15 days | ⭘ 1 fois par mois  Once per month | ⭘ 1 fois par trimestre  Once every 3 months |
| --- | --- | --- |
| ⭘ 1 seule fois sur l’année  Once per year | ⭘ 2 fois sur l’année  Twice per year | ⭘ Aucune fois  Never |

9. Si l’on vous propose de participer à une recherche médicale, quelles sont les évaluations/explorations que vous refuseriez ? (Cochez autant de croix que vous le souhaitez)

If you are offered to participate in medical research, what assessments/explorations would you refuse? (Check as many boxes as you want)

| 🞏 Examens du souffle  Breathing / lung function | 🞏 IRM du coeur/thorax  Heart / chest MRI |
| --- | --- |
| 🞏 Scintigraphie pulmonaire  Pulmonary scintigraphy | 🞏 Test de marche durant 6 minutes  6 minute walking test |
| 🞏 Prise de sang veineuse  Blood test (from vein in arm) | 🞏 Tests cutanés allergologiques  Allergic skin tests |
| 🞏 Scanner des poumons  CT scan of the lungs | 🞏 Evaluations psychologiques  Psychological assessments |
| 🞏 Test d’effort sur bicyclette  Exercise test on bicycle | 🞏 Électrocardiogramme  Electrocardiogram |
| 🞏 Gaz du sang artériels (prélèvement de sang dans l’artère du poignet)  Arterial blood gases (blood taken from an artery in your wrist) | |
| 🞏 Bronchoscopie (examen, sous anesthésie locale, permettant d’examiner les bronches à l’aide d’une caméra insérée par le nez ou par la bouche)  Bronchoscopy (examination, under local anesthesia, allowing the bronchi to be examined using a camera inserted through the nose or  mouth) | |
| 🞏 Remplir des questionnaires portant sur votre santé  Fill out questionnaires about your health | |
| 🞏 Cathétérisme cardiaque droit (cathéter introduit dans une veine du bras ou au pli de l'aine, pour mesurer les pressions artérielles dans l'artère pulmonaire)  Right heart catheterization (catheter inserted into a vein in the arm or at the fold of the groin, to measure arterial pressures in the  pulmonary artery) | |
| 🞏 Test de provocation à la méthacholine (dépistage de l’asthme : inhalation d’un produit mimant les symptômes de l'asthme)  Methacholine provocation test (asthma screening: inhalation of a product mimicking asthma symptoms) | |
| 🞏 Remplir quotidiennement à domicile un carnet de bord de vos symptômes  Complete a daily symptom diary at home | |
| 🞏 Mesurer votre souffle à domicile à l’aide d’un petit appareil  Measure your breath at home using a small device | |

Tournez la page SVP

Please turn the page

10. Si l’on vous propose de tester un nouveau médicament, seriez-vous d'accord ?

If you're offered to test a new medicine, would you agree?

| - Avant commercialisation ?  Before market approval? | ⭘ Oui  Yes | ⭘ Non  No | - Après commercialisation :  After market approval? | ⭘ Oui  Yes | ⭘ Non  No |
| --- | --- | --- | --- | --- | --- |
| Si vous avez répondu au moins 1 oui à la question 10 : accepteriez-vous de tester ce nouveau médicament…  If you answered at least 1 yes to question 10: would you accept to test this new medicine… | | | | | |
| … par voie inhalée ?  … by inhalation? | ⭘ Oui  Yes | ⭘ Non  No | … par voie orale ?  … orally? | ⭘ Oui  Yes | ⭘ Non  No |
| … par voie sous cutané ?  … by subcutaneous route? | ⭘ Oui  Yes | ⭘ Non  No | … par voie intra-veineuse ?  … intravenously? | ⭘ Oui  Yes | ⭘ Non  No |

11. Accepteriez-vous de participer à une recherche médicale sans indemnisation ?

Would you accept to participate in medical research without compensation?

⭘ Oui ⭘ Non

Yes No

12. Quelles sont les institutions avec lesquelles vous accepteriez de participer à une recherche clinique ? (réponses multiples possibles)

Which institutions would you agree to participate in clinical research with? (multiple responses possible)

| 🞏 Laboratoires pharmaceutiques  Pharmaceutical laboratories | 🞏 Instituts de recherche publique (INSERM, CNRS)  Public research institutes (INSERM, CNRS) |
| --- | --- |
| 🞏 Centres Hospitaliers Universitaires  University Hospitals | 🞏 Cliniques médicales privées  Private medical clinics |
| 🞏 Centres Hospitaliers Généraux  General Hospitals |  |

Assurez-vous que vous avez répondu à toutes les questions, merci pour votre collaboration.

Make sure you have answered all the questions, thanks for your cooperation.
