# Supplementary material for: Patient distrust in pharmaceutical companies: an explanation for women under-representation in respiratory clinical trials?
Source: BMC Med Ethics. 2020 Aug 13;21:72. doi: 10.1186/s12910-020-00509-y (PMC7424561; doi:10.1186/s12910-020-00509-y)
Supplement: Supplementary file 1 — Additional file 1. Questionnaire PROTOACCEPT - French/English bilingual version. [file 12910_2020_509_MOESM1_ESM.zip › PROTOACCEPT1_english_questionnaireR5.docx]

**This English translation of the questionnaire is provided for information only.**

**The validated questionnaire is the French questionnaire**

Madam, Sir,

We would like you to take a few minutes to complete this medical research questionnaire. This questionnaire has 4 pages, it is anonymous and there are no right or wrong answers.

| Age:……… ………….Years | Sex: ⭘ Male ⭘ Female |
| --- | --- |

Number of people living in your home/residence: ………

The highest level of education you reached (check one box)

| ⭘ *Primary school*  ⭘ *Middle school / Junior High School*  ⭘ *High school* | ⭘ *University or equivalent*  ⭘ *Other ………………………………………..* |
| --- | --- |

Your employment situation (check one or more boxes)

| 🞏  *Independent or self-employed*  🞏  *Civil servant*  🞏  *Stable employment (Permanent contract)*  🞏  *Precarious/short-term employment (fixed-term contract, interim, internship)*  🞏  *Non-compensated job seeker*  🞏  *Job seeker receiving benefits* | 🞏  *Home-maker*  🞏  *Retired*  🞏  *Active Solidarity Income recipient*  🞏  *Disabled adult allowance*  🞏  *Disabled worker allowance*  🞏  *Student*  🞏 *Other ……………………………….* |
| --- | --- |

1 *Do you know the name of the illness for which you are consulting today?*

⭘ *Yes*  ⭘ *No*

If yes, what is its name? ………………………………..…………………………………….

Please turn the page

2.  *Is this your first consultation for your respiratory illness?*

⭘ *Yes*  ⭘ *No*

3.  *Have you ever participated in a medical research protocol?*

⭘ *Yes*  ⭘ *No*

4. Si un médecin vous proposait de participer à un protocole de recherche médicale, quelle serait votre première réaction ?

If a doctor suggested that you participate in a medical research protocol, what would be your first reaction?

⭘ *Yes*  ⭘ *No* ⭘ *I would like to think about it*

5. *In the table below:*

A - *Please read everything first, then choose and tick 3 proposals that seem to you the most characteristic of "Medical Research". Do not tick more than 3 boxes.*

B - *Then, in the remaining proposals, please choose and tick 3 proposals which seem to you the least characteristic of "Medical Research". Do not tick more than 3 boxes.*

(Please respect all of the instructions)

| Medical Research is above all… | Most characteristic | Least characteristic |
| --- | --- | --- |
| … a possibility for patients to have the most recent treatments | 🞏 | 🞏 |
| … a matter of money | 🞏 | 🞏 |
| … a way to make progress in science | 🞏 | 🞏 |
| … a way to use the sick and their illnesses | 🞏 | 🞏 |
| … an economic investment for a country | 🞏 | 🞏 |
| … a risk to patients and their health | 🞏 | 🞏 |
| …better medical care for the sick | 🞏 | 🞏 |
| … an issue at stake for pharmaceutical companies | 🞏 | 🞏 |
| … hope of healing for the sick | 🞏 | 🞏 |
|  | You must tick three | You must tick three |

Please turn the page

6.  *Here is a list of reasons that may explain why you would accept to participate in medical research. Please indicate to what extent you agree or disagree with all of these reasons by checking the appropriate box.*

| I would accept because…. | Strongly disagree | Disagree | Neither agree nor disagree | Agree | Strongly agree |
| --- | --- | --- | --- | --- | --- |
| … it’s useful for my current health | ⭘ | ⭘ | ⭘ | ⭘ | ⭘ |
| … I want to advance science | ⭘ | ⭘ | ⭘ | ⭘ | ⭘ |
| … my family encourages me to do it | ⭘ | ⭘ | ⭘ | ⭘ | ⭘ |
| … I will see my doctor more often | ⭘ | ⭘ | ⭘ | ⭘ | ⭘ |
| … it’s compensated | ⭘ | ⭘ | ⭘ | ⭘ | ⭘ |
| … I will feel better taken care of | ⭘ | ⭘ | ⭘ | ⭘ | ⭘ |
| … I want to heal | ⭘ | ⭘ | ⭘ | ⭘ | ⭘ |
| … I've got time | ⭘ | ⭘ | ⭘ | ⭘ | ⭘ |
| … it's hard to say no to a doctor | ⭘ | ⭘ | ⭘ | ⭘ | ⭘ |
| … I trust my doctor | ⭘ | ⭘ | ⭘ | ⭘ | ⭘ |
| … it’s useful for my future health | ⭘ | ⭘ | ⭘ | ⭘ | ⭘ |
| … it’s useful for those who have the same illness as me | ⭘ | ⭘ | ⭘ | ⭘ | ⭘ |
| ... someone close to me said positive things about it | ⭘ | ⭘ | ⭘ | ⭘ | ⭘ |

Please turn the page

7. *Here is a list of the reasons that may explain why you would refuse to participate in medical research. Please indicate to what extent you agree or disagree on all of these reasons by checking the appropriate box.*

| I would refuse because…. | Strongly disagree | Disagree | Neither agree nor disagree | Agree | Strongly agree |
| --- | --- | --- | --- | --- | --- |
| … It's useless for my current health | ⭘ | ⭘ | ⭘ | ⭘ | ⭘ |
| … I don't like going to the hospital | ⭘ | ⭘ | ⭘ | ⭘ | ⭘ |
| … I don't like going to the doctor | ⭘ | ⭘ | ⭘ | ⭘ | ⭘ |
| … I’m wary of the situation | ⭘ | ⭘ | ⭘ | ⭘ | ⭘ |
| … I don’t trust pharmaceutical companies | ⭘ | ⭘ | ⭘ | ⭘ | ⭘ |
| … I don't like medical exams | ⭘ | ⭘ | ⭘ | ⭘ | ⭘ |
| … I don't have enough time | ⭘ | ⭘ | ⭘ | ⭘ | ⭘ |
| ... someone close to me said negative things about it | ⭘ | ⭘ | ⭘ | ⭘ | ⭘ |
| … I live too far away | ⭘ | ⭘ | ⭘ | ⭘ | ⭘ |
| … It's difficult to come in | ⭘ | ⭘ | ⭘ | ⭘ | ⭘ |
| … I don't want to be a guinea pig | ⭘ | ⭘ | ⭘ | ⭘ | ⭘ |
| … It is expensive to come in often | ⭘ | ⭘ | ⭘ | ⭘ | ⭘ |
| … I risk getting a placebo | ⭘ | ⭘ | ⭘ | ⭘ | ⭘ |

Please turn the page

8.  *How much time could you give to medical research over a period of one year? (Only one answer possible)*

| ⭘  *Once every 15 days* | ⭘ *Once per month* | ⭘  *Once every 3 months* |
| --- | --- | --- |
| ⭘  *Once per year* | ⭘  *Twice per year* | ⭘  *Never* |

9.  *If you are offered to participate in medical research, what assessments/explorations would you refuse? (Check as many boxes as you want)*

| 🞏  *Breathing / lung function* | 🞏  *Heart / chest MRI* |
| --- | --- |
| 🞏  *Pulmonary scintigraphy* | 🞏  *6 minute walking test* |
| 🞏  *Blood test (from vein in arm)* | 🞏  *Allergic skin tests* |
| 🞏  *CT scan of the lungs* | 🞏  *Psychological assessments* |
| 🞏  *Exercise test on bicycle* | 🞏  *Electrocardiogram* |
| 🞏 *Arterial blood gases (blood taken from an artery in your wrist)* | |
| 🞏  *Bronchoscopy (examination, under local anesthesia, allowing the bronchi to be examined using a camera inserted through the nose or*  mouth) | |
| 🞏  *Fill out questionnaires about your health* | |
| 🞏  *Right heart catheterization (catheter inserted into a vein in the arm or at the fold of the groin, to measure arterial pressures in the pulmonary artery)* | |
| 🞏  *Methacholine provocation test (asthma screening: inhalation of a product mimicking asthma symptoms)* | |
| 🞏  *Complete a daily symptom diary at home* | |
| 🞏  *Measure your breath at home using a small device* | |

Please turn the page

10. *If you're offered to test a new medicine, would you agree?*

| -  *Before market approval?* | ⭘ Yes | ⭘ No | -  *After market approval?* | ⭘ Yes | ⭘ No |
| --- | --- | --- | --- | --- | --- |
| If you answered at least 1 yes to question 10: would you accept to test this new medicine… | | | | | |
| … by inhalation? | ⭘ Yes | ⭘ No | … orally? | ⭘ Yes | ⭘ No |
| … by subcutaneous route? | ⭘ Yes | ⭘ No | … intravenously? | ⭘ Yes | ⭘ No |

11.  *Would you accept to participate in medical research without compensation?*

⭘ *Yes*  ⭘ *No*

12.  *Which institutions would you agree to participate in clinical research with? (multiple responses possible)*

| 🞏 *Pharmaceutical laboratories* | 🞏 *Public research institutes (INSERM, CNRS)* |
| --- | --- |
| 🞏 *University Hospitals* | 🞏 *Private medical clinics* |
| 🞏 *General Hospitals* |  |

Make sure you have answered all the questions, thanks for your cooperation.
